# Supplementary material for: Spiritual well-being as a protective factor for endothelial dysfunction in clinically healthy adults
Source: PLoS One. 2025 Dec 26;20(12):e0339676. doi: 10.1371/journal.pone.0339676 (PMC12742740; doi:10.1371/journal.pone.0339676)
Supplement: Attachment — Submitted filename: RESPONSE TO REVIEWERS.docx [file pone.0339676.s002.docx]

**Response to Reviewers – Manuscript Title: "Spiritual Well-Being as a Protective Factor for Endothelial Dysfunction in Clinically Healthy Adults: A Cross-Sectional Analysis"**

Dear Editors,

Dear Academic Editor and Reviewers,

We would like to thank you for the thorough and thoughtful review of our manuscript. Below, we provide a detailed point-by-point response to the comments raised, indicating the changes made in the revised manuscript.

### ****Academic Editor / Journal Office****

**1. Reference verification:**

**Response:** We reviewed all citations for accuracy. Reference #8 was corrected to a validation study conducted in Brazil, and reference #10 was replaced by a source including cut-off scores for the FACIT-Sp scale.

**2. Justify dichotomization of independent variables.**

**Response:** We appreciate the reviewer’s observation regarding the choice to dichotomize the independent variables (spiritual well-being, anxiety, and depression). As noted, we initially analyzed these variables in their continuous forms. The hypothesis of multicollinearity between FACIT and PHQ-9 (r = 0.7, p < 0.001), PHQ-9 and GAD (r = 0.50, p < 0.05), and PHQ-9 and GAD (r = 0.45, p < 0.005) was not rejected. Therefore, we proceeded by considering only the FACIT as the independent variable to assess its association with endothelial function.

Correlation Matrix: P**earson correlation coefficients between FACIT-Sp, GAD-7, and PHQ-9 total scores (n = 148).**

**3. Clarify definition of endothelial dysfunction (ED).**

**Response:** We standardized the definition of ED throughout the manuscript as FMD <10%, in accordance with established international and national studies.

**4. Address inconsistencies in statistical reporting.**

**Response:** We appreciate the reviewer’s observation. The mention of t-tests and chi-squared tests in the Methods section was indeed an oversight on our part. These tests were initially considered during the early stages of the analysis plan; however, as the study progressed, we opted for a more comprehensive statistical approach, employing logistic regression and discriminant analysis to address our research questions in a multivariate framework.

This advanced modeling strategy allowed us to simultaneously adjust for potential confounders and assess the combined discriminatory power of the predictors, which rendered the use of separate t-tests and chi-squared tests unnecessary. We have now removed the reference to these tests in the Methods section to ensure consistency and clarity.

**5. Address reduced power in secondary analysis.**

**Response:** We explicitly acknowledge the reduced statistical power of the secondary model after excluding participants with anxiety or depression. This limitation has been discussed in the revised Limitations section.

**6. Data availability and STROBE compliance.**

**Response:** Supporting data have been included in the revised supplementary files, and the manuscript has been checked for compliance with STROBE guidelines. The STROBE checklist is also submitted with this revision.

**7. Avoid causal language and overstated conclusions.**

**Response:** We carefully revised the Discussion and Conclusion sections to avoid causal language. Statements implying causality were rephrased to reflect the cross-sectional design, emphasizing the exploratory and correlational nature of our findings.

**8. Address residual variance and generalizability.**

**Response:** We added a paragraph to discuss the proportion of unexplained variance in the regression models. The decrease in explained variance in the secondary model was also addressed, reinforcing that mental health variables play a significant role and that other unmeasured factors may also influence ED.

### ****Reviewer #1****

**1. Add specific research questions at the end of the introduction.**
**Response:** We thank the reviewer for this helpful suggestion. We have added the specific research questions to the end of the Introduction section, clearly enumerated as follows:

(1) Was spiritual well-being (SWB) associated with endothelial dysfunction (ED)?
(2) Did this association persist after excluding individuals with anxiety or depressive symptoms?
(3) Which SWB dimensions—peace, meaning, or faith—showed the strongest association with ED?

**2. Strengthen the methodology regarding sampling method and adequacy.**
**Response:** We revised the "Materials and Methods" section to describe the convenience sampling approach and recruitment process in greater detail, including participant sources (e.g., students, staff, and patient relatives). We also included a justification for sample adequacy based on previous literature and added results from a post hoc power analysis confirming sufficient statistical power (β > 0.80).

**3. Include validity and reliability coefficients of measurement tools.**
**Response:** We have added internal consistency coefficients (Cronbach’s alpha) and validation references for all scales used (FACIT-Sp, PHQ-9, and GAD-7) in the "Measures" subsection. In the present study, internal consistency reliability coefficients were high for the FACIT-Sp (Cronbach’s alpha [α] = 0.862), PHQ-9 (α = 0.814), and GAD-7 (α = 0.890).The manuscript now includes information confirming their psychometric properties in Brazilian populations.

**4. Emphasize clinical relevance of SWB effects beyond anxiety and depression.**
**Response:** As recommended, we have enhanced the Discussion to emphasize that SWB remained a significant predictor of preserved endothelial function even after excluding individuals with anxiety or depression. This highlights its potential independent effect and clinical relevance beyond traditional psychological dimensions.

**5. Highlight the independence of peace and meaning dimensions from faith.**
**Response:** This important point was incorporated into the Discussion and reinforced in the section on discriminant analysis. We emphasized that inner peace and life purpose significantly differentiated individuals with and without ED, whereas the faith dimension did not, underscoring the cross-cultural and secular applicability of SWB.

**6. Note the limited generalizability due to sample size and setting.**
**Response:** We fully agree. The limitations section has been expanded to acknowledge the modest sample size and its restriction to a Brazilian population, cautioning against broad generalizations. We also call for larger, multi-center studies to validate our findings.

### ****Reviewer #2****

**1. Include prior studies linking spirituality and cardiovascular outcomes.**
**Response:** We thank the reviewer for these references. We incorporated the suggested citations (O'Riordan et al., 2025; Lucchetti, 2025; Cottrell-Daniels et al.) into the Introduction and Discussion to contextualize our findings within the broader literature on spirituality and cardiovascular health.

**2. Include information on the context of spirituality in Brazil.**

**Response:** We expanded the Discussion to include a brief overview of spirituality/religiosity in Brazil, referencing national surveys and academic initiatives. We noted the cultural relevance and increasing institutional support for spirituality in health promotion.

**3. Discuss barriers to address spirituality in clinical settings.**

**Response:** We integrated a paragraph discussing barriers such as lack of training, limited time, and clinicians’ discomfort. We cited recent studies showing that although physicians recognize the relevance of spirituality, many feel unprepared to address it in clinical practice.

**4. Acknowledge study strengths.**

**Response:** The strengths section has been revised to highlight the use of validated instruments, rigorous clinical screening, and the novel integration of psychological and spiritual variables in cardiovascular risk assessment.

**5. Clarify clinical implications.**

**Response:** The Discussion now more clearly outlines potential clinical implications, emphasizing the exploratory nature of our findings and the importance of integrating SWB in a culturally sensitive and non-dogmatic manner into preventive strategies.

**** Furthermore, we undertook a comprehensive re-examination of the dataset and the database with methodological accuracy and scientific rigor. This process led to minor adjustments in certain numerical values; however, the overall interpretation, significance, and conclusions of the study remain unchanged.**

**As requested, the database has been attached to the submission.**

We sincerely thank the reviewers for their constructive suggestions, which significantly improved the rigor and clarity of our manuscript. All changes made in response to these comments are marked in the revised version submitted.

Respectfully,

Sincerely,
**André Casarsa, MD, MSc** and Prof. Sergio Schmidt PhD (Corresponding author)

On behalf of all co-authors

Department of Internal Medicine University Hospital Gaffrée Guinle – UNIRIO

Federal University of the State of Rio de Janeiro
slschmidt@terra.com.br
