# Supplementary material for: Spiritual well-being as a protective factor for endothelial dysfunction in clinically healthy adults
Source: PLoS One. 2025 Dec 26;20(12):e0339676. doi: 10.1371/journal.pone.0339676 (PMC12742740; doi:10.1371/journal.pone.0339676)
Supplement: Attachment — Submitted filename: RESPONSE TO REVIEWERS 2.docx [file pone.0339676.s003.docx]

**Response to Reviewers – Manuscript Title: "Spiritual Well-Being as a Protective Factor for Endothelial Dysfunction in Clinically Healthy Adults"**

Dear Editors,

Dear Academic Editor and Reviewers,

We would like to thank you for the thorough and thoughtful review of our manuscript. Below, we provide a detailed point-by-point response to the comments raised, indicating the changes made in the revised manuscript.

### ****Academic Editor / Journal Office****

**1.Cut-off for SWB (FACIT-Sp):** **In the revised manuscript, it is unclear whether the cut-off of 36 was determined based on your sample or directly adopted from McClain et al. (≥3 “quite a bit” vs <3 “somewhat” or lower). This cut-off is not a clinically or psychometrically validated threshold, but a data-driven value specific to McClain’s sample. Without previously validated cut-offs (e.g., ROC-based thresholds), the rationale for dichotomizing SWB remains weak. Authors should either justify this choice based on their own data distribution or consider more robust alternatives.**

**Response:** We appreciate the reviewer’s thoughtful observation. In response, we revised our analytic strategy to enhance methodological rigor and to directly address the issue raised.

While our initial approach was conceptually inspired by McClain et al. (2003), we acknowledge that the threshold used in their study was developed within an oncology population and lacks external psychometric validation, particularly for application in cardiovascular or clinically healthy populations. As no validated cut-off for the FACIT-Sp currently exists for predicting endothelial dysfunction or related cardiovascular outcomes in non-clinical samples, we recognize that the dichotomization approach could introduce unnecessary limitations.

Accordingly, we reanalyzed all models using the FACIT-Sp total score as a continuous variable. This decision reflects both statistical and conceptual considerations. Statistically, treating spiritual well-being as continuous preserves the variability inherent in the construct, improves power, and avoids potential loss of information and misclassification bias associated with arbitrary dichotomization. Conceptually, spiritual well-being is widely recognized as a dimensional construct, and prior literature supports its use as a continuous predictor in models examining psychological and physical health outcomes, including in cardiovascular contexts [1,2,3,4].

This updated approach aligns with best practices in quantitative research, especially in the absence of clinically validated cut-offs, and allows for more nuanced interpretation of the association between spiritual well-being and endothelial function. All references to the dichotomized SWB variable were removed, and the revised manuscript now consistently reports results based on continuous treatment of the FACIT-Sp total score.

1. Peterman AH, Fitchett G, Brady MJ, Hernandez L, Cella D. Measuring spiritual well-being in people with cancer: The functional assessment of chronic illness therapy—Spiritual Well-being Scale (FACIT-Sp). Ann Behav Med. 2002;24(1):49–58. doi:10.1207/S15324796ABM2401_06
2. Edmondson D, Park CL, Blank TO, Fenster JR, Mills MA. Deconstructing spiritual well-being: Existential well-being and HRQOL in cancer survivors. Psychooncology. 2008;17(2):161–169. doi:10.1002/pon.1221
3. Cotton S, Puchalski CM, Sherman SN, Mrus JM, Peterman AH, Feinberg J, et al. Spirituality and religion in patients with HIV/AIDS. J Gen Intern Med. 2006;21(S5):S5–13. doi:10.1111/j.1525-1497.2006.00642.x
4. Park CL, Edmondson D, Fenster JR, Blank TO. Meaning making and psychological adjustment following cancer: The mediating roles of growth, life meaning, and restored just-world beliefs. *J Consult Clin Psychol.* 2008;76(5):863–875. doi:10.1037/a0013348

**2. Justification for dichotomization:** **It is unclear why a linear regression model with continuous predictors was added in the revised manuscript. Please clarify the rationale for including this model. If it was intended to justify the dichotomization of SWB, it is not apparent how it accomplishes this. Consider whether the linear regression is necessary or whether the main focus should remain on the logistic regression with ED as the outcome. If Authors decide to maintain this model thanks to a strong rationale, it should be also clarified why the same predictors were treated as continuous in the linear regression (continuous endothelial function outcome) and dichotomized in the logistic regression (including ED as a binary outcome). Moreover, Authors stated that they proceeded by considering only SWB as the independent variable due to collinearity; however, PHQ and GAD are still included in the logistic regression model (Table 1). Multicollinearity alone does not justify dichotomizing a continuous variable, especially in the absence of previously validated cut-offs. Explain why dichotomization was preferred over keeping SWB continuous and addressing collinearity through standard methods (e.g., variable exclusion, combination, or other methods).**

**Response:** We appreciate the reviewer’s thoughtful comments and the opportunity to clarify our analytical approach. In the previous version of the manuscript, a linear regression model using flow-mediated dilation (FMD%) as a continuous outcome was included as an exploratory analysis. However, upon further reflection, we recognize that this model did not directly contribute to the primary aims of the study, which focused on identifying predictors of clinically relevant endothelial dysfunction. Furthermore, the inclusion of the linear regression introduced inconsistencies in the treatment of predictor variables and may have raised interpretative challenges by suggesting alternative modeling paths without a clearly defined theoretical rationale. Accordingly, we have removed the linear regression analysis from the manuscript to ensure greater conceptual and statistical coherence.

Regarding the treatment of spiritual well-being (FACIT-Sp), we have revised our analytical strategy to model this construct as a continuous predictor in all inferential analyses. While our original approach followed the cut-off proposed by McClain et al. (2003), we acknowledge that this threshold was derived from a specific oncology population and lacks external psychometric validation in clinically healthy or cardiovascular samples. In line with current best practices in quantitative health research, we now preserve the full variability of the FACIT-Sp scale to avoid the loss of statistical power and the risk of arbitrary categorization, which could obscure meaningful associations.

In a prior response, we referred to potential collinearity concerns as one reason for restricting the initial model to SWB. However, to properly evaluate this issue, we performed diagnostic analyses using variance inflation factor (VIF) and tolerance values for all covariates included in the final logistic regression model. The results indicated no evidence of problematic multicollinearity, with all VIFs below 2.0 and tolerances above 0.5. Based on these findings, we confirmed that multicollinearity was not a limiting factor in our model specification and thus did not require dichotomization of SWB or exclusion of additional predictors.

This updated modeling approach ensures a more robust interpretation of our findings, strengthens the alignment between the theoretical construct and the statistical framework, and directly addresses the reviewer’s concern about collinearity and the limitations of arbitrary dichotomization.

**3. Assessment of multicollinearity:** **Reporting correlations alone is insufficient. A correlation >0.70 does not automatically indicate problematic multicollinearity. Please provide VIF and tolerance values for all predictors to substantiate any claims of collinearity. Attention should be given to any inconsistencies (e.g., correlation between PHQ and SWB reported as >0.70 in your reply, but ~0.50 in the figure). Note: the presence of multicollinearity does not automatically justify dichotomization.**

**Response:** We appreciate the reviewer’s important observation regarding the assessment of multicollinearity. In our previous response, we referenced bivariate correlations as a preliminary indication of shared variance among predictors. However, we agree that correlation coefficients alone are insufficient for diagnosing problematic multicollinearity in regression models.

To address this issue more rigorously, we conducted a formal collinearity diagnostic analysis using Variance Inflation Factor (VIF) and tolerance values for all independent variables included in the logistic regression model. The results revealed no evidence of problematic multicollinearity: all VIF values were below 2.0, and all tolerance values exceeded 0.5, which are well within commonly accepted thresholds (VIF < 5.0 and tolerance > 0.2) for retaining predictors in multivariable models.

Additionally, we reviewed our previously reported correlation coefficients and confirmed that the correlation between SWB (FACIT-Sp) and PHQ-9 was approximately –0.50, not above –0.70 as erroneously stated in our prior reply. We apologize for this oversight and have corrected the text accordingly.

As a result, we concluded that multicollinearity does not pose a threat to the validity of our models and did not serve as the basis for any variable transformation or exclusion. In line with this updated assessment and the concerns raised in Item 2, we have removed the dichotomization of the FACIT-Sp score and now model spiritual well-being as a continuous predictor in all inferential analyses.

We believe these steps provide a more robust and transparent statistical approach and fully address the reviewer’s concerns regarding the evaluation and implications of multicollinearity.

### ****Reviewer #1****

### The manuscript is in a highly developed state after revision. Thank you for considering the suggestions of all the reviewers. The introduction, methods (sample, data analysis), and discussion have become particularly cautious, especially regarding the mediating effect of SWB on cardiovascular health. From my perspective, the limitations are also satisfactory. With this revision, I believe the manuscript can contribute to the literature. Some spelling errors, punctuation, and comma usage appear differently in the tables. There are also parts where ED is not used. Please check the final proof.

### ****Reviewer #2****

**The manuscript has been improved and can be published in the present version.The authors have adequately addressed my comments raised in a previous round of review and I feel that this manuscript is now acceptable for publication**

**Response:** We sincerely thank both reviewers for their generous feedback and for recognizing the improvements made in the revised version of our manuscript.

To **Reviewer #1**, we are particularly grateful for the acknowledgment of the refinements in the introduction, methods, and discussion, as well as for the thoughtful remarks regarding clarity and rigor. As recommended, we carefully reviewed the entire manuscript for consistency in terminology (e.g., “ED”), spelling, punctuation, and formatting within the tables and figures. All identified issues have been corrected in the final version.

To **Reviewer #2**, we truly appreciate your positive assessment and support for publication. Your earlier suggestions played a key role in improving the clarity and relevance of our findings.

Finally, we extend our sincere thanks to the *PLOS ONE* editorial team for overseeing this process with fairness and professionalism. We are honored by the opportunity to publish in such a respected and widely read journal, and we hope this work will contribute meaningfully to ongoing discussions at the intersection of spirituality, mental health, and cardiovascular research.

Sincerely,
**André Casarsa, MD, MSc** and Prof. Sergio Schmidt PhD (Corresponding author)

On behalf of all co-authors

Department of Internal Medicine University Hospital Gaffrée Guinle – UNIRIO

Federal University of the State of Rio de Janeiro
slschmidt@terra.com.br
