# Supplementary material for: Spiritual well-being as a protective factor for endothelial dysfunction in clinically healthy adults
Source: PLoS One. 2025 Dec 26;20(12):e0339676. doi: 10.1371/journal.pone.0339676 (PMC12742740; doi:10.1371/journal.pone.0339676)
Supplement: Attachment — Submitted filename: RESPONSE TO REVIEWERS 3.docx [file pone.0339676.s004.docx]

**Response to Reviewers – Manuscript Title: "Spiritual Well-Being as a Protective Factor for Endothelial Dysfunction in Clinically Healthy Adults"**

Dear Editors,

Dear Academic Editor and Reviewers,

We would like to thank you for the thorough and thoughtful review of our manuscript. Below, we provide a detailed response to the comments raised, indicating the changes made in the revised manuscript.

### ****Academic Editor / Journal Office****

**1.** **The authors have done an excellent job revising the manuscript. There remains one minor but important issue. In the new regression model, statements (including in the abstract) suggesting that SWB is the “strongest predictor” overreach the data. Indeed, some variables (e.g., major depressive episodes) showed larger coefficients and odds ratios. This needs to be addressed to accurately reflect the results.**

**As a possible way to revise the text, the authors may consider rephrasing the sentences in line with the first research question, focusing on the fact that SWB is significantly inversely associated with ED, independent of other covariates. This would convey the key finding without overstating the relative strength of the predictor.**

**Response:** We thank the reviewer for the thoughtful and constructive comment. We agree that statements referring to spiritual well-being (SWB) as the “strongest predictor” overstated the findings. As suggested, we have revised the abstract and the main text to ensure that the results are accurately represented.

Specifically, we removed expressions implying comparative predictive strength and rephrased the sentences to emphasize that SWB was significantly and inversely associated with endothelial dysfunction (ED), independent of other covariates, which is consistent with our primary research question. These changes avoid overinterpretation and better reflect the observed effect sizes relative to other variables in the model.

We appreciate the reviewer’s guidance, which improved the clarity and precision of our manuscript.

Finally, we extend our sincere thanks to the *PLOS ONE* editorial team for overseeing this process with fairness and professionalism. We are honored by the opportunity to publish in such a respected and widely read journal, and we hope this work will contribute meaningfully to ongoing discussions at the intersection of spirituality, mental health, and cardiovascular research.

Sincerely,
**André Casarsa, MD, MSc** and Prof. Sergio Schmidt PhD (Corresponding author)

On behalf of all co-authors

Department of Internal Medicine University Hospital Gaffrée Guinle – UNIRIO

Federal University of the State of Rio de Janeiro
slschmidt@terra.com.br
